# Possible Role of Mitochondrial DNA Mutations in Chronification of Inflammation: Focus on Atherosclerosis

**DOI:** 10.3390/jcm9040978

**Published:** 2020-04-01

**Authors:** Alexander N. Orekhov, Nikita N. Nikiforov, Ekaterina A. Ivanova, Igor A. Sobenin

**Affiliations:** 1 Laboratory for Angiopathology, Institute of General Pathology and Pathophysiology, 125315 Moscow, Russia; 2 Laboratory of Infection Pathology and Molecular Microecology, Institute of Human Morphology, 117418 Moscow, Russia; 3 Centre of Collective Usage, Institute of Gene Biology, Russian Academy of Sciences, 34/5 Vavilova Street, 119334 Moscow, Russia; nikiforov.mipt@googlemail.com; 4 Institute of Experimental Cardiology, National Medical Research Center of Cardiology, 121552 Moscow, Russia; 5 Department of Basic Research, Institute for Atherosclerosis Research, 121609 Moscow, Russia; 6 Laboratory of Medical Genetics, Institute of Experimental Cardiology, National Medical Research Center of Cardiology, 121552 Moscow, Russia; igor.sobenin@gmail.com

**Keywords:** atherosclerosis, chronification of inflammation, defective mitophagy, innate immunity, mitochondrial dysfunction, modified LDL

## Abstract

Chronification of inflammation is the process that lies at the basis of several human diseases that make up to 80% of morbidity and mortality worldwide. It can also explain a great deal of processes related to aging. Atherosclerosis is an example of the most important chronic inflammatory pathology in terms of public health impact. Atherogenesis is based on the inflammatory response of the innate immunity arising locally or focally. The main trigger for this response appears to be modified low-density lipoprotein (LDL), although other factors may also play a role. With the quick resolution of inflammation, atherosclerotic changes in the arterial wall do not occur. However, a violation of the innate immunity response can lead to chronification of local inflammation and, as a result, to atherosclerotic lesion formation. In this review, we discuss possible mechanisms of the impaired immune response with a special focus on mitochondrial dysfunction. Some mitochondrial dysfunctions may be due to mutations in mitochondrial DNA. Several mitochondrial DNA mutations leading to defective mitophagy have been identified. The regulatory role of mitophagy in the immune response has been shown in recent studies. We suggest that defective mitophagy promoted by mutations in mitochondrial DNA can cause innate immunity disorders leading to chronification of inflammation.

## 1. Introduction

Chronification of inflammation is relevant to many human disorders and a subject of active current research that aims to answer the numerous questions remaining to date. If we could explain why the immune response can be prolonged in time, creating sites of chronic inflammation, it would help to clarify the mechanisms of diseases that account for up to 80% of current morbidity and mortality, as well as the mechanisms involved in aging. In this review, we discuss the role of the immune response disturbance at the level of mitochondrial dysfunction, in particular, defective mitophagy. Being a specialized form of autophagy, mitophagy clears the cell from dysfunctional mitochondria that can also be dangerous for the cells. It is known that mitophagy defects may be caused by some mitochondrial DNA (mtDNA) mutations. We describe the possible input of mtDNA mutations in the chronification of inflammation that can cause defects in mitophagy and, as a consequence, cause a massive release of pro-inflammatory factors and an unresolving inflammatory reaction.

Atherosclerosis is a convenient model to study chronification of inflammation. Atherosclerotic lesions develop in the arterial wall locally, and even focally. Early stages of the disease development are characterized by prominent local differences between the adjacent areas of the arterial wall in terms of inflammatory activation. Comparison of such areas may reveal the cells, molecules and signaling pathways that are responsible for inflammation chronification.

Despite the common understanding that atherosclerosis is an inflammatory disease, use of general anti-inflammatory drugs, such as non-steroidal anti-inflammatory drugs (NSAIDs) could not be proven beneficial for treatment of the disease. Some of the suggested approaches were doomed to failure, since they impacted targets not related to atherosclerosis [1,2,3]. It is therefore important to better understand the relationship between inflammation and the atherosclerotic process, discussion of which became one of the topics of this review. At the same time, some of the therapies that are protective against atherosclerosis, such as statins, also have anti-inflammatory properties [4].

In this review, we discuss cellular mechanisms of atherosclerosis development. During the last decade, studies of atherosclerosis pathogenesis have been conducted mostly on transgenic animal models. However, the cellular composition and architecture of the arterial wall in animals and humans differs significantly, as does the structure of atherosclerotic lesions [5,6]. One of the possible mechanisms leading to chronification of inflammation is mitochondrial dysfunction, which is tightly related to increased oxidative stress and cell death. We hereby present the hypothesis that defective mitophagy (clearance of dysfunctional mitochondria) caused by mtDNA mutations plays a central role in the transition of normal inflammatory reaction of the innate immunity to chronic inflammation, which can be observed in atherosclerosis.

## 2. Atherosclerosis as Inflammatory Disease

Atherosclerosis is currently regarded as an inflammatory disease, which is dependent on the chronic inflammation at all stages of its development. The disease can affect any artery, but the most dangerous lesions evolve in the aorta, coronary artery, arteries supplying brain and kidneys, and lower limb vessels. Atherosclerotic lesions develop locally, and even focally, progressively increasing in size and obstructing the blood vessel lumen, sometimes leading to a complete obstruction of the blood flow.

Local inflammatory processes accompanying atherosclerotic lesion development are quite distinct by their mechanisms from the classical symptoms of inflammation, such as pain and erythema. Histological analysis of atherosclerotic lesions reveals local infiltration and activation of monocytes/macrophages and cytokine release followed by tissue reparation and remodeling. Such local inflammation driven by activated immune cells also result in the local healing response. Resolved inflammation is generally believed to result in diffuse intimal thickening which is normal for an adult artery. Thanks to modern imaging techniques, not only plaque morphology, but also disease activity can be observed in patients’ vessels affected by atherosclerosis [7]. Therefore, at the tissue level, atherosclerosis can be clearly identified as an inflammatory process.

**Figure 1:**
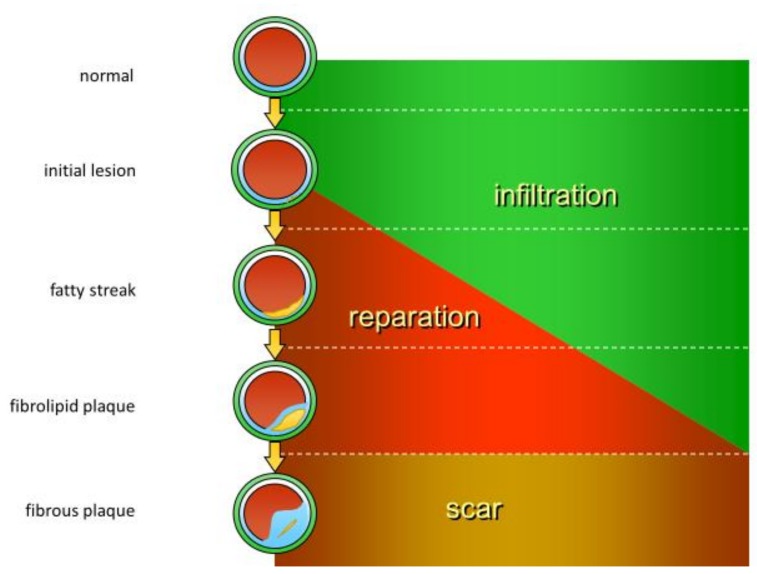
Phases of inflammation in relation to atherosclerotic lesion development. The commonly accepted course of atherosclerotic lesion progression generally corresponds to the consecutive stages of the inflammatory response, with the formation of stable, fibrous plaque being comparable to scarification.

Histological features of inflammation include three consecutive stages: infiltration, reparation, and scar formation, which can also be observed during atherosclerotic lesion formation (Figure 1). Early stages of atherosclerotic lesion development involve local activation and loss of function of the vessel endothelium, which allows for the increased endothelial permeability for lipoprotein particles and recruitment of circulating immune cells. The emerging atherosclerotic plaques are therefore characterized by local lipid infiltration into the arterial wall corresponding to the swelling phase of the inflammatory process. Later stages include continuing lipid accumulation that leads to the formation of so-called fatty streak further progressing to fibrolipid plaque, which presents with signs of local extracellular matrix synthesis and alteration of the blood vessel wall cellular content. This stage is accompanied by the reparative processes, including cell proliferation and extracellular matrix synthesis. In case of successful resolution of inflammation, a scar is formed, which, in the context of atherosclerotic plaque development, corresponds to the formation of a stable fibrous cap that separates the lipid core from the blood vessel lumen. Different types of immune cells have been detected both in human atherosclerotic lesion specimens and in animal models of atherosclerosis [8,9,10]. Moreover, it was demonstrated that the numbers of immune cells present in atherosclerotic lesions are prominently increased in comparison to the surrounding unaffected tissues [8], highlighting the active involvement of innate immunity mechanisms in atherosclerotic lesion development.

At the organism level, increased levels of inflammation markers have long been known to be associated with atherosclerosis development [11,12] and have been evaluated as cardiovascular risk predictors [13]. However, attention should be paid not to confuse the atherosclerotic process, occurring locally, or even focally, with a generalized inflammatory condition, since therapeutic approaches for treating these conditions will be different. For instance, NSAIDs inhibiting cyclooxygenase-2 (COX2) were shown not to reduce, but to increase the cardiovascular risk by affecting the putative protective function of prostacyclin [14].

The use of statins for reducing cardiovascular risk have taken a firm place in current clinical practice. It was shown that beneficial effects of statins are not limited to lipid metabolism normalization, but are actually pleiotropic. Importantly, statins possess beneficial effects on vascular inflammation, protecting against atherosclerotic plaque development [4]. Blocking of the interleukin 1 beta (IL-1β) inflammatory pathway has been extensively evaluated for reducing cardiovascular risk. In the CANTOS trial (Canakinumab Anti-inflammatory Thrombosis Outcomes Study), a monoclonal antibody targeting IL-1β canakinumab was shown to reduce the level of the inflammatory marker C-reactive protein (CRP) and to reduce the cardiovascular risk, without influencing blood lipid profile [15]. However, while clinical parameters related to atherosclerosis improved in patients receiving canakinumab, no information is available so far on its possible effect on atherosclerotic plaque development. Following the promising results with canakinumab, other anti-inflammatory agents targeting the IL-1β have been proposed, including the small molecules colchicine and methotrexate [16]. While methotrexate at low doses failed to show significant anti-inflammatory effects and to protect against cardiovascular events [17], colchicine appeared to be more promising. A recent study has showed its potential for plaque stabilization in a rabbit model of atherosclerosis [18]. However, more studies are needed to evaluate its effects on atherosclerotic plaque development in humans. Therefore, the need for novel anti-atherosclerotic therapies acting at the arterial wall level still persists, and numerous current studies are aimed at identifying new therapeutic targets and disease modifiers. The success of this search will depend on our understanding of the mechanisms of atherosclerotic plaque formation.

## 3. Cellular Mechanisms of Atherogenesis

**Figure 2:**
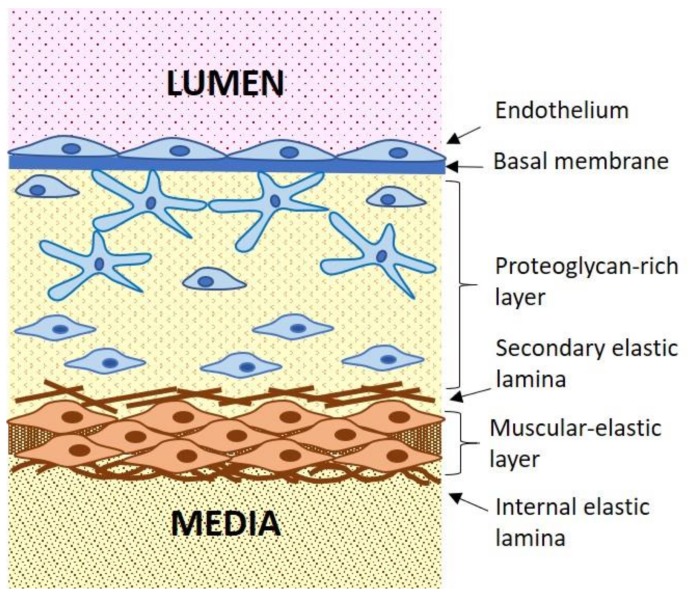
Schematic presentation of the arterial intima. Proteoglycan-rich layer contains several cell types, including stellate pericyte-like cells and modified smooth muscular cells with reduced contractility.

Atherosclerotic lesion development takes place in the arterial wall intima, the innermost layer of the arterial wall (Figure 2). The intimal layer of the adult large arteries is relatively thick and is characterized by a complex architecture and heterogeneous cell content.

Arterial wall intima is separated from the blood vessel lumen by a monolayer of endothelial cells (ECs) that are located on the basal membrane. The endothelial layer plays an important function in the arterial wall maintenance, regulating the penetration of cellular and chemical components of circulating blood into the arterial wall [19]. The endothelial layer is characterized by certain heterogeneity [20]. Alongside the regular ECs with typical morphology, the endothelial layer can contain clusters of large polynuclear cells. Typically, these cells are absent from the vessels of young organisms, and appear during the period that corresponds to atherogenesis initiation. Moreover, such clusters are observed more frequently within the areas susceptible to atherosclerotic lesion development. These areas often experience disturbed laminar blood flow that occurs near blood vessel bends, bifurcations, and branching. The heterogeneity of the vessel endothelial layer may in part explain the focal nature of atherogenesis in the arteria wall. It is possible that clusters of giant polynuclear cells have increased permeability for circulating lipoprotein and disturbed endothelial function, which plays a crucial role at the initial stages of atherosclerosis development.

Beneath the basal membrane, there is a thick proteoglycan-rich layer, also called elastic-hyperplastic layer, which is populated by various cell types [21]. In the proximity of the endothelial layer, the proteoglycan-rich layer may contain macrophages (approximately 3%-5% of the cell population), dendritic cells (0.3%) and other immune cells that are rarely observed in deeper layers of the arterial intima. Macrovascular pericytes that have a distinct stellar morphology represent up to 25%–30% of the cellular population of this layer [22]. These cells are connected with each other through specialized cell contacts on their long, branching processes, forming a three-dimensional cellular network within the proteoglycan-rich layer (Figure 2). This cellular network appears to serve as a second line of defense after the endothelial layer within the arterial wall [22]. The stellate-shaped, pericyte-like cells can execute phagocytic functions alongside with macrophages and can participate in the immune response by producing pro-inflammatory cytokines [23]. Moreover, they can develop a capacity for antigen processing and presentation, like dendritic cells [24,25]. The immune functions of pericytes are less pronounced than those of professional immune cells, such as macrophages and dendritic cells. However, because the number of pericytes present in the arterial wall intima is 10 to 100 times greater than that of immune cells, their input in the innate immune response is significant. The most abundant cell type of the proteoglycan-rich layer, taking up to 70% of the total cell population, are smooth muscular cells. These smooth muscular cells are, however, different from the typical smooth muscular cells present in the media, since they have reduced capacity for constriction and increased synthetic activity. They can be therefore be referred to as modified smooth muscular cells [26]. The proteoglycan-rich layer is separated from the adjacent muscular-elastic layer by the secondary elastic lamina. The outer part of the muscular elastic intima is populated by the typical vascular smooth muscular cells (VSMCs) that are also present in the media.

Atherosclerotic lesion development is accompanied by profound changes of the cellular contents of the intimal layer of the arterial wall [24]. First, local increase of cellularity is observed. Among the cell types, abundantly present in the growing lesion are macrophages that actively take part in the local inflammatory process. The cellular network is disrupted, and cellular contacts are lost, leading to reduced intercellular communication. Pericyte-like cells, macrophages and modified smooth muscular cells accumulate lipids, giving rise to so-called foam cells that have cytoplasm filled with undegraded lipid droplets. Foam cell formation is one of the earliest and most prominent features observed during atherosclerotic lesion development at the cellular level.

**Figure 3:**
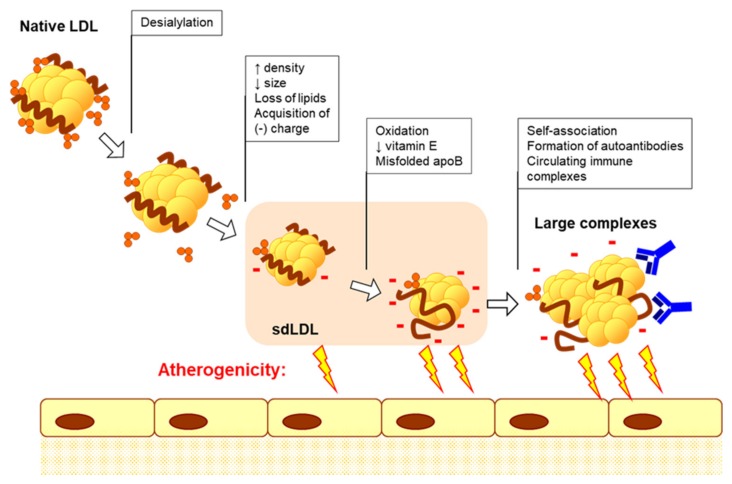
Cascade of multiple atherogenic modification of low-density lipoprotein (LDL). Multiple atherogenic modifications of LDL particles have been detected in human blood plasma: desialylation was the first event, followed by loss of free cholesterol and cholesterol esters, phospholipids and triglycerides, increase in particle density and decrease in its size; next, negative charge of particles was increased, leading to the formation of electronegative LDL fraction, in which misfolded apolipoprotein B (apoB) was reported; at later stages, increased oxidation and decreased antioxidant content were observed; finally, large highly atherogenic complexes can be formed due to self-association of modified LDL particles and the formation of autoantibodies.

Atherogenic modified LDL is the primary source of lipids accumulating in foam cells [27]. Lipoprotein particles circulating in a patient’s blood undergo atherogenic modifications that alter the protein, lipids and glycoconjugate moieties [28]. Importantly, multiple atherogenic modification of LDL involves several stages, starting from desialylation, and ending with oxidation and formation of large, highly atherogenic complexes [29]. The stepwise atherogenic modification of LDL particles involves gradual reduction of sialic acid contents, changes of particle physical-chemical properties, including increasing density and acquisition of negative charge, and increasing capacity for complex formation. Finally, modified LDL particles may induce formation of autoantibodies (Figure 3). Circulating LDL-containing immune complexes are characterized by high atherogenicity, hence the ability to induce intracellular lipid accumulation when added to cultured cells [29].

**Figure 4:**
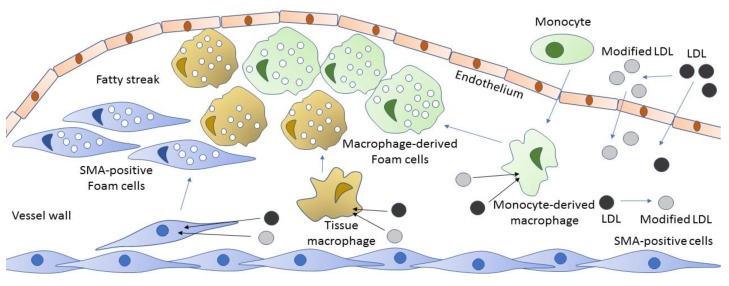
Involvement of subendothelial cells in foam cell formation. From the blood flow, both LDL and modified LDL enter the vessel wall, where they can be internalized by macrophages, pericytes and vascular smooth muscle cells (vascular smooth muscle a-actin (SMA)-positive cells) via scavenger receptors or by phagocytosis or pinocytosis. These macrophages and SMA-positive cells with the taken-up lipid contents in their cytoplasm become foam cells. Adapted from [37], with permission.

**Figure 5:**
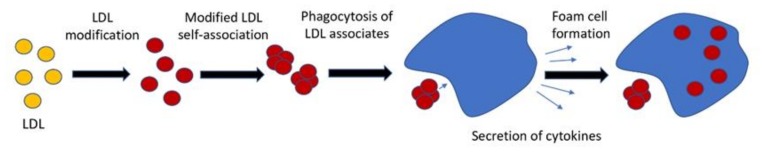
Role of inflammatory cytokines in triggering foam cell formation. Adapted from [37], with permission.

Native LDL interacts with a specialized LDL receptor (LDLR), and is normally internalized via receptor-mediated endocytosis, being destined for lysosomal degradation [30]. This process ensures that lipids taken up by the cell are utilized in cellular catabolic processes, and the excess lipids are excreted from the cells via corresponding efflux mechanisms [31]. It was shown that, in high concentrations, native LDL can be taken up by pinocytosis leading to intracellular cholesterol accumulation [32]. Under normal conditions, cholesterol efflux mediated by high-density lipoprotein (HDL) and transporter proteins protect the arterial wall from excessive lipid accumulation. However, if the balance between the accumulation of intracellular lipids and the outflow of excess fat is violated, lipids are deposited in the form of fat dots and droplets, which leads to the formation of foam cells when fat droplets fill the cytoplasm (Figure 4). Large LDL associates that contain modified LDL stimulate phagocytic activity of cells, including subendothelial macrophages and pericytes. Comparable in their size with certain pathogens, large lipoprotein associates are likely to trigger the innate immune reaction and initialize unspecific clearance processes. Up-regulated phagocytosis, in turn, leads to the increased production of pro-inflammatory cytokines that attract monocytes and other immune cells to the emerging site of atherosclerotic lesion. Different research groups have shown that pro-inflammatory cytokines promote intracellular lipid accumulation induced by atherogenic modified LDL [33,34,35,36,37]. Moreover, in some cases, intracellular lipid accumulation is induced under inflammatory conditions even when modified LDL is not present [37]. Therefore, foam cell formation is likely to be caused in the first place not by the intracellular lipid accumulation, but by the immune response triggered by the interaction of cells with modified LDL (Figure 5).

**Figure 6:**
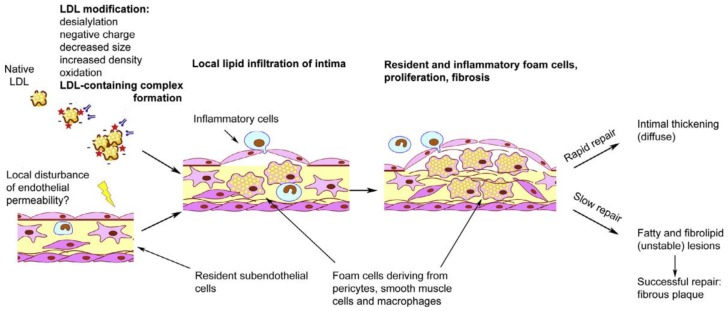
Schematic overview of initiation of atherosclerotic lesion formation. Adopted from [40], with permission.

Intracellular lipid accumulation is likely to be responsible for the loss of intercellular contacts and the destruction of the network of pericyte-like cells in the subendothelial space [38]. This process is accompanied by the increase of proliferative capacity of cells and the elevation of extracellular matrix synthesis [38,39]. These processes are characteristic of the reparative phase of the immune response and tissue remodeling after the resolution of inflammation. Under favorable circumstances, such reparation occurs rather quickly and results in the formation of a moderate thickening of the intimal layer of the arterial wall at the lesion site. Such focal lesions accumulate with age and can be observed in the arterial wall specimens in the form of diffuse intimal thickening (Figure 6). This diffuse thickening is distinct from the atherosclerotic lesion, and can be considered normal for the arteries of an adult organism [26]. In cases when normal resolution of focal inflammation is not possible, the acute inflammatory phase can transform into a chronic process, leading to a true atherosclerotic lesion development. Therefore, innate immune response can be regarded as a trigger of foam cell formation, while disturbances in the normal immune response lead to chronification of inflammation [40]. It can be concluded that innate immune response and chronification of sterile inflammation together play a key role in atherogenesis.

## 4. Innate Immunity Response and Cellular Mechanisms of Inflammation

The innate immunity response is evolutionarily the oldest system of organism defense against pathogen invasion. It can be triggered by microbial infection and intrusion of other foreign matters, and is represented by cellular immune response, primarily executed by tissue macrophages [41]. The main inducers of the innate immunity response are pathogen-associated molecular patterns (PAMPs) and damage-associated molecular patterns (DAMPs) that are produced by invading pathogens and damaged cells and tissues respectively. The large self-associates of modified LDL can serve as DAMPs, in a similar manner to other particles of corresponding size. Therefore, atherogenic modified LDL can be regarded as atherosclerosis-specific stimulus for sterile (non-infectious) inflammation. Macrophages, the key players of the innate immunity response, are responsible for pathogen destruction and elimination, as well as for the recruitment of other immune cells to the evolving inflammation site [42]. The first part of this task is performed through macrophage phagocytic activity, while the second is performed through stimulation of pro-inflammatory cytokine secretion that accompanies phagocytosis. In case of microbial infection, the inflammatory response is induced by PAMPs that are recognized through Toll-like receptors (TLRs) and cytoplasmic nucleotide-binding oligomerization domain (NOD)-like receptors (NLRs) [41,43,44]. Notably, these receptors were also shown to be involved in the interaction of modified LDL with cells [45,46,47,48,49]. The signaling induced by the interaction of PAMP or DAMP with innate immune cell receptors triggers the activation of nuclear factor (NF)-κB-mediated production and secretion of pro-inflammatory cytokines, which further leads to the recruitment of the effector cells and their inflammatory action in response to infection [41,50]. The NF-κB signaling pathway appear to be the main mediator of the inflammatory signals in the innate immunity response [51,52]. Moreover, this pathway is also responsible for the production of pro-inflammatory cytokines and activation of the inflammasome [53]. Inflammasome activation is a central event in the inflammatory response, which stimulates further production of pro-inflammatory cytokines, including IL-1ß and IL-18, and the recruitment of the effector immune cells [54,55]. In certain pathologies, inflammasome activation is disrupted leading to chronic inflammatory disorders [53,55,56,57].

Of special interest is the link between inflammasome activation and mitochondrial dysfunction, which appears to be especially important in atherosclerosis [58,59]. Several research groups have demonstrated the involvement of inflammasomes-mediated signaling regulation for mitochondrial function [60,61,62]. It is currently clear that mitochondrial dysfunction and damage is involved in NLRP3 inflammasomes activation [60]. Dysfunctional mitochondria serve as an important source of DAMPs and as the inducers of cells oxidative damage and death implicated in several chronic human pathologies [63].

## 5. Innate Immunity and Mitochondrial Dysfunction

Mitochondria are semi-autonomous organelles derived from ancient endosymbiotic prokaryotic organisms that retain some important features of their bacterial ancestors [64]. Importantly, mitochondria possess their own circular genome (mitochondrial DNA, mtDNA), which encodes a large part, but not all, mitochondrial proteins. A cell can contain from tens to hundreds of mitochondria, each of them containing its own copy of the genome. If all mtDNA copies are equal, they are called homoplasmic, while co-existence within the same cell of different mtDNA variants is called heteroplasmy. Mutations in mtDNA occur 5-15 times more frequently than in the nuclear DNA due to a less reliable mitochondrial DNA reparation system. These mutations are also inherited by the maternal line and then multiply by dividing mitochondria containing mutant mtDNA. This determines the high level of mtDNA variability, as well as the gradual accumulation of somatic mutations of mtDNA with aging [65,66,67,68,69]. MtDNA mutations can play a significant, or even a key, role in pathological processes, affecting the genes encoding components of mitochondrial electron transport chains or mitochondrial tRNA genes. Although respiratory chain enzymes are mainly encoded by the nuclear genome, nevertheless, mtDNA defects can lead to a decrease in enzyme activity and loss of functionality, as well as errors in the assembly of respiratory complexes and, as a result, to overall mitochondrial dysfunction, which contributes to the accelerated development of pathology.

Mitochondria realize fission and fusion independently of cell division, and can increase or reduce their numbers within the cell [70]. The inner membrane of the mitochondria contains phospholipid cardiolipin, which is unique to prokaryotic membranes [71]. It is therefore no wonder that dysfunctional mitochondria act as an important DAMPs source via exposing mtDNA and cardiolipin, and can, under pathological circumstances, be recognized as a pathogen by the innate immunity system. Exposure of the mitochondrial DAMPs usually accompanies tissue damage [72,73].

Mitochondrial homeostasis may be impaired due to the infection that leads to mtDNA release and excessive reactive oxygen species (ROS) production. These processes are aimed at destroying the invading pathogens, but can be dangerous to the host cells and tissues as well. Mitochondrial damage triggers NLRP3, which promotes the activation of caspase-1 and generation of the pro-inflammatory IL-1β and IL-18 [55,74]. These processes are activated by cardiolipin transfer from the inner- to outer-mitochondrial membrane and its association with NLRP3 [75]. Moreover, mitochondrial stimulation of inflammation and inflammation-induced mitochondrial dysfunction can promote each other, forming a vicious circle [76].

Mitochondrial quality control is executed by several mechanisms that help monitor mitochondrial damage, reduce mitochondrial stress, and ensure selective removal of defective mitochondrial proteins or entire damaged organelles [77,78]. An effective pathway towards removal of dysfunctional organelles is mitophagy, a selective form of autophagy, in which damaged or dysfunctional mitochondria undergo lysosomal degradation. Mitophagy is an important element of mitochondrial quality control [79]. Defective mitophagy causes a pronounced accumulation of damaged mitochondria and excessive inflammation caused by pro-inflammatory cytokines [80]. Mitophagy, in turn, is dependent on a correct balance between mitochondrial fission and fusion processes [81]. Mitochondrial fission ensures effective mitophagy, while fusion contributes to biogenesis and restoration of the organelles. These processes are dependent on several proteins that have been extensively studied during the recent years. Interestingly, it was shown that mitochondrial dynamics are altered with aging, which is especially important in the myocardium [81]. Inhibition of the key regulator of mitochondrial fission, Drp1, abolished mitophagy and exacerbated heart failure in a mouse model [82]. At the same time, another study conducted in mice showed that inhibition of Drp1-mediated mitochondrial fission alleviated cardiac dysfunction in diabetic mice [83]. These observations highlight the importance of the balance between mitochondrial fission and fusion for maintaining mitochondrial homeostasis, which is especially important for cardiac function. Thus, mitophagy controls a self-limiting machinery that supports homeostasis. These and other results demonstrate the key role of mitochondria in the transmission of signals of the innate immunity and position mitophagy as a key regulatory mechanism that preserves tissue homeostasis by limiting excessive inflammation [84].

## 6. Mitochondrial Dysfunction and mtDNA Mutations in Chronic Pathologies

Being a central player in maintaining cellular energy homeostasis and regulators of apoptosis and cell survival, mitochondria are naturally implicated in numerous human disorders. However, their role in chronification of inflammation appears to be especially important for chronic human disorders that are known to be associated with inflammatory processes. Interesting parallels can be drawn between human diseases that involve chronic inflammation and presence of mitochondrial dysfunction and mtDNA mutations. The existence of such parallels also makes mitochondrial dysfunction an attractive point of therapeutic intervention.

The link between mitochondrial dysfunction and atherosclerosis has been the subject of extensive research [85]. Besides cardiovascular disease, several chronic human pathologies are known to be associated with mitochondrial dysfunction. Mitochondria play an important role in tumor development. The uncontrolled tumor growth is dependent on the suppressed immune response accompanied by chronic inflammatory conditions. Mutations in mtDNA that occur in tumor cells can lead to mitochondrial dysfunction and cancer progression [86]. These dysfunctions are associated with disturbed cellular energy metabolism, which is a characteristic feature of oncogenesis [87,88]. It is currently well known that mitochondrial dysfunction in cancer affects the inflammatory response and cell homeostasis and also contributes to the progression of cancer [89,90].

Chronic obstructive pulmonary disease (COPD) is associated with aberrant inflammatory reactions in the lungs, which also involves mitochondrial dysfunction. In particular, mitochondrial genes MAVS and NLRX1 were shown to play a role in COPD pathogenesis [91,92,93]. In patients with COPD, mitochondrial dysfunction, decreased density of mitochondria, and altered mtDNA content were found in the skeletal muscle cells [94,95]. Moreover, mitochondrial fission was shown to be associated with the destruction of lung tissue [96]. The alveolar macrophage functions of patients with COPD were shown to be impaired due to mitochondrial dysfunction [97]. Other chronic pulmonary diseases, such as pulmonary fibrosis, asthma, cystic fibrosis, and pulmonary hypertension were also shown to be associated with mitochondrial dysfunction [98,99].

Among the many processes involved in aging, impaired mitochondrial function and alteration of mitochondrial count have been identified as prominent players [100]. Recently, the concept of inflammaging, which means pro-inflammatory conditions characteristic of the aging organism, has emerged [101,102,103]. There is currently no doubt that chronic inflammation is a major risk factor for morbidity and mortality in the elderly population [102]. Throughout life, stress factors disrupt critical mitochondrial functions such as redox reactions, oxidative phosphorylation, and mitophagy [104]. The aging process is accelerated by mitochondrial dysfunction resulting from defects in mtDNA repair mechanisms. Mitochondrial function gradually decreases with age leading to the increase of mitochondrial DAMP release. As a consequence, innate immune response stimulation contributes to the onset and progression of chronic inflammatory diseases associated with aging [105,106].

Age-related accumulation of mtDNA mutations has been observed in many studies, and the possible role of mtDNA mutations in aging was widely considered [65,66,67,68,69]. Study of the functional significance of mtDNA mutations became possible with the development of cytoplasmic hybrid (cybrid) models. Cybrids are cell lines that are produced as a result of fusion of a whole cell with a cytoplast of enucleated cells (platelets) that contain mitochondria bearing mtDNA mutations of interest. Using cybrids carrying several point mutations in mtDNA, Li et al. demonstrated the significant enrichment of these mutations in aging mitochondria [107]. The results of their study suggest that the aging-associated mtDNA mutations result in mitochondrial dysfunction through altering the oxidative phosphorylation machinery. Furthermore, mtDNA mutations were shown to be among the most important causes of hearing loss [108]. Homoplasmic mutations m.1555A>G and m.1494C>T were found to be associated with hearing loss worldwide. Moreover, five mutations associated with hearing loss have been identified in the tRNA genes: m.7445A>G, m.7472insC, m.7505T>C, m.7510T>C, and m.7511T>C. Other mutations are mainly localized to the genes encoding tRNA and proteins. Cybrid lines carrying these mutations presented with mitochondrial dysfunction features. In a study of cybrid cells carrying the deafness-associated mutation tRNA^His^ m.12201T>C, Gong et al. found that overexpression of the human mitochondrial histidyl-tRNA synthetase gene corrected the mitochondrial dysfunction caused by this mutation [109]. These results not only contribute to our understanding of the pathophysiology of mitochondrial diseases, but also create a scientific basis for the development of therapeutic interventions for mitochondrial disorders.

A recent study conducted by our group has reported a relationship between mitochondrial functions and atherosclerosis-associated mtDNA mutations investigated on cybrid cell lines carrying various variants of the mitochondrial genome obtained from atherosclerotic patients [110]. Earlier studies of mtDNA obtained from the leukocytes of atherosclerotic patients revealed a correlation between certain mtDNA mutations and atherosclerosis [111,112,113]. Such mutations could be homoplasmic (absence or presence of the mutation) or heteroplasmic (different proportions of mutant allele). Another recent study identified variants of mtDNA that uncoupled oxidative phosphorylation: m.del562G, m.1555A>G, m.14459G>A, and m.14846G>A. Variants of mtDNA that sustained ATP synthase activity were m.3256C>T, m.12315G>A, and m.13513G>A [110]. The m.14459G>A mtDNA variant was associated with a higher basal rate of oxygen consumption. None of the mtDNA variants affected the rate of oxygen consumption upon the addition of succinate and KCN. The m.1555A>G and m.14846G>A mtDNA variants were associated with the opposite effect. These variants therefore may have a protective function.

Mutations of the mtDNA associated with conditions that increase the risk of atherosclerosis development, such as hypertension, type 2 diabetes and obesity have been described [114,115,116,117].

Functional activity of cells involved in the immune-inflammatory response is highly dependent on correct functioning of the mitochondria. Impairment of mitochondrial respiration may result in both normal aging of blood vessels and in various cardiovascular diseases, including atherosclerosis, heart failure, and the formation of aneurysm [118]. Mitochondrial mtDNA mutations are not specifically linked to certain pathologies in most cases. This is no wonder, taking into account that mitochondrial dysfunction may manifest itself in different pathological processes. For instance, mtDNA mutations identified as associated with atherosclerosis [119], have also been detected in such pathologies as gastric carcinoma [120], hearing impairment and loss [121,122,123], mitochondrial encephalomyopathy, lactic acidosis and stroke-like episodes (MELAS) [124], type 2 diabetes [125], acute myocardial infarction [126], encephalopathy [127], Li syndrome (hereditary encephalomyopathy), Wolff-Parkinson-White syndrome (preexcitation syndrome), cardiomyopathy [128], hereditary Leber’s optic atrophy [129], and other diseases [130,131].

## 7. Mosaicism of Atherosclerosis-Associated mtDNA Mutations in the Vascular Wall

**Figure 7:**
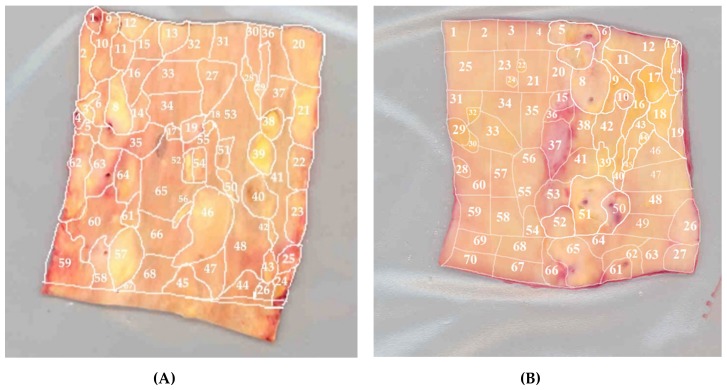
Morphological mapping of the aorta samples. Presented are two examples of morphological mapping of the aortic wall: (**A**) and (**B**). Segments of the vascular wall were divided according to morphological characteristics into 68 (**A**) and 70 (**B**) regions containing atherosclerotic lesions of varying severity (fatty infiltration, fatty streak, lipofibrous plaque, fibrous plaque) or unaffected tissue. These and other aorta samples were further analyzed for the mutational burden in mtDNA. Adapted from [119] with permission.

Several heteroplasmic mtDNA mutations positively or negatively correlating with the extent of atherosclerotic lesions in human arteries have been identified in circulating cells, atherosclerotic arteries, and other tissues. These mutations are located in the mitochondrial genes encoding rRNA12S, tRNA-Leu (UUR recognition codon), tRNA-Leu (CUN recognition codon), subunits of 1, 2, 5, and 6 NADH-dehydrogenase, and cytochrome B [113,132]. Heteroplasmy levels of atherosclerosis-associated mtDNA mutations were determined in grossly normal and atherosclerotic segments of morphologically mapped aortic walls [119]. In total, 265 normal and atherosclerotic tissue loci of five human aorta samples were investigated (Figure 7). Every aortic sample was divided according to morphological characteristics into loci with different types of atherosclerotic lesions: fibrous plaque, fibrolipid plaque, fatty streak, or fatty infiltration. Uninvolved loci and loci of different types of atherosclerotic lesions significantly differed in the content and composition of atherosclerosis-associated mutations. Moreover, different loci of the aortic intima, including grossly normal and affected by atherosclerotic lesions of different stages differed in the heteroplasmy level of the mutant mtDNA variant.

**Figure 8:**
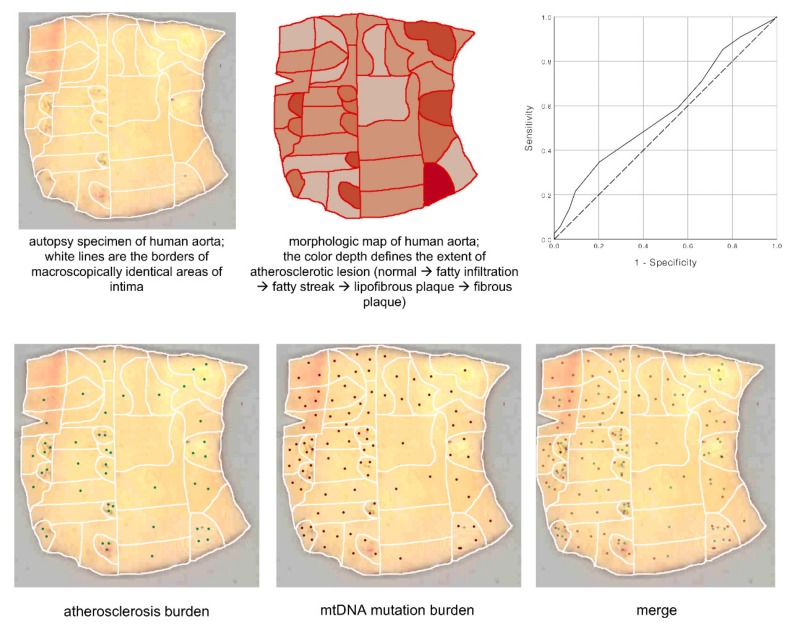
Co-localization of mtDNA mutation and atherosclerosis in human aortic intima. A significant correlation between mtDNA mutation burden and atherosclerosis burden was observed: *r* = 0.131, *p* = 0.034 (Spearman’s rho). The area under received operating characteristic (ROC)-curve for mtDNA mutation burden accounted for 0.587 (95% confidence interval (CI) 0.506–0.668, *p* = 0.041), the positive actual state was the presence of advanced atherosclerotic lesions.

The focal nature of the atherosclerotic lesion distribution within the arterial wall is clearly seen in Figure 7. Such mosaicism may correspond to previously described clusters containing dysfunctional endothelial cells with impaired permeability. The distribution of mtDNA mutations among various types of atherosclerotic lesions, as well as in unaffected areas in the human aortic intima, is shown on Figure 8. The authors also reported a correlation between the accumulation of mtDNA mutations and the severity of atherosclerotic lesions. The more pronounced the lesion, the more mtDNA mutations were detected in the corresponding area. In areas with the most advanced lesions, a maximal accumulation of mtDNA mutations was observed, i.e., these areas presented with the highest mtDNA mutation burden.

The observed mosaicism in mtDNA mutation distribution may have different explanations. First, it may reflect the focal nature of atherosclerotic lesion initiation, which preferentially occurs in the sites of local disturbance of the endothelial function and increased permeability. Second, due to the fact that even non-involved loci are significantly differed from each other by the content and composition of atherosclerosis-associated mutations [119], the mosaicism of mtDNA mutations distribution may partly explain the focal distribution of atherosclerotic lesions. It cannot be ruled out that the observed local accumulation of mtDNA mutations is due to a local accumulation of leukocytes carrying pro-atherogenic mutations recruited to the inflammation site. Finally, it can be assumed that the mosaicism of atherosclerosis is the result of local changes in both endothelium and subendothelial intima.

## 8. Innate Immunity and mtDNA Mutations

A study conducted by our group has shown that mtDNA mutations are related to the degree of monocyte activation [133]. In this study, pro-inflammatory activation of monocytes isolated from the blood of subjects with asymptomatic atherosclerosis correlated with atherosclerosis-associated mtDNA mutations, in particular, homoplasmic m.1811A>G and m.9477G>A. At least three other heteroplasmic mutations, m.14459G>A, m.1555A>G, and m.12315G>A, also correlated with pro-inflammatory activation of monocytes. It was suggested that some mutations may alter monocyte-derived macrophage activation in atherosclerosis.

**Figure 9:**
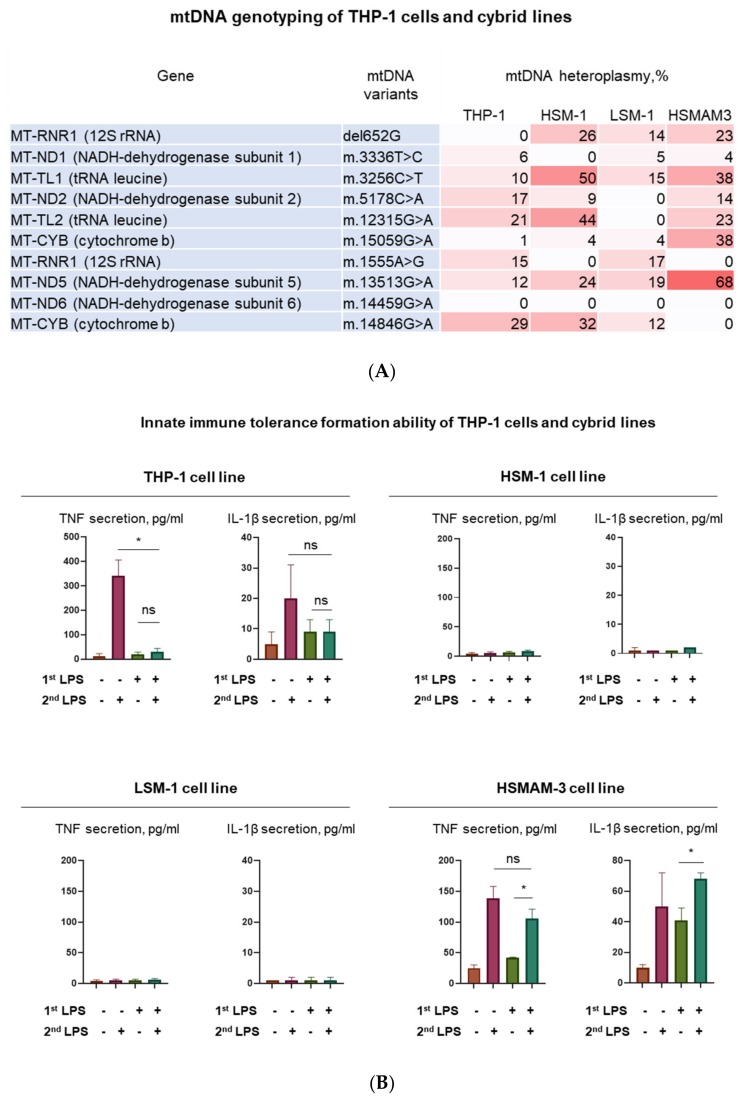
Ability of THP-1 cells and cybrid lines to form innate immune tolerance. (**A**) MtDNA genotyping of THP-1 cells and cybrid lines was performed and mtDNA heteroplasmy index was measured for 10 mtDNA mutations. (**B**) Innate immune tolerance formation ability of THP-1 cells and cybrid lines. First, 1 µg/mL of lipopolysaccharides (LPS) was added to the cells cultured in suspension in RPMI medium (10% FBS) for 16 h (1st LPS). Then, cells were washed by sterile PBS and fresh RPMI medium with or without 1 µg/mL of LPS was added for 4 h (2nd LPS). Finally, the secretion of tumor necrosis factor (TNF) and IL-1β was evaluated by ELISA. A statistical analysis of the results of three independent experiments was carried out using the IBM SPSS Statistics 21 software package. The significance of *p* < 0.05 according to the results of t-test for paired samples is marked with an asterisk. Ns - no significance, i.e., *p* > 0.05.

Three cybrid lines carrying mitochondrial mutations associated with atherosclerosis (see above) were used to evaluate the response to inflammatory stimulation with lipopolysaccharides (LPS). Cybrid lines were created from macrophage-like cells of the THP-1 line, ρ-0 cells, that do not have functional mtDNA, fused with platelets isolated from different patients. Thus, each cybrid cell line contained a nuclear genome of THP-1 cells and the patient’s mitochondrial genome. Each cybrid line had the mitochondrial genotype of a single patient (Figure 9A). The left panel of Figure 9 shows the results of mtDNA genotyping of the studied cell lines. Genotyping was carried out on ten known mitochondrial mutations associated with atherosclerosis. The left column of each table shows the mtDNA genes in which mutations were detected. The middle column presents mtDNA variants. Heteroplasmic indices are presented in the right column and reflect the proportion of mitochondrial DNA containing the corresponding mutation in the studied cells. Genotyping of native THP-1 cells, as well as three cybrid lines HSM-1, LSM-1, and HSMAM-3 obtained by transplantation of mtDNA obtained from atherosclerotic patients into THP-1 cells. Heteroplasmy was detected in native THP-1 cells for some mutations, which however did not exceed 30% in any of the mutations. It was also found that all the studied cybrids were different in the heteroplasmy profile for the studied mutations, both among themselves and in comparison with THP-1.

The right panel of Figure 9 shows the different responses of the three cybrids and their ability to form immune tolerance to LPS. In the case of the intact THP-1 line (control), an adequate response to LPS stimulation was observed, which manifested itself in an increase in the secretion of pro-inflammatory cytokines after the first stimulation and no effect after the second stimulation (Figure 9B). This is a manifestation of immune tolerance or training in innate immunity [134,135]. For the HSM-1 and LSM-1 cybrid lines, the secretion of tumor necrosis factor (TNF) and IL-1β did not exceed 10 pg / mL, which indicates that these cybrids are insensitive to LPS. A special place among the cybrid lines is occupied by HSMAM-3. Cells of this line responded to LPS stimulation. In addition, upon repeated stimulation, the cells responded by secretion of cytokines. This indicates the inability of HSMAM-3 cells to form immune tolerance.

Thus, cells differing in the mitochondrial genome and carrying atherogenic mutations were dramatically different in their immune response from intact THP-1 cells. Two cybrid lines did not respond to inflammatory stimulation, but the third line not only responded to the first but also to the second stimulation, i.e., this cybrid line lacked tolerance of innate immunity. Presence of such cells in the focus of inflammation will complicate the resolution of the inflammatory response and will contribute to the chronification of inflammation.

## 9. Mitophagy and mtDNA Mutations

MtDNA mutations may also be involved in mitophagy impairment, and, as a consequence, insufficient clearance of dysfunctional mitochondria. To ensure mitophagy, several molecular mechanisms that preserve cellular and tissue homeostasis are activated and coordinated [136]. Violations of mitophagy regulation can lead to serious changes in mitochondrial metabolism and, ultimately, to unresolved inflammation of tissue. Various microbial components were found to be targeting mitophagy therefore allowing the infection to bypass the defense of the innate immunity. Mitophagy plays a key role in the regulation of signaling pathways of inflammation. In addition, mitophagy regulates the response to mitochondrial danger, for example, the appearance of mtDNA in the cytosol, which contributes to an increase in the inflammatory response. Defective mitophagy disrupts the immune response at the level of secretion of inflammatory cytokines, which leads to impaired normal functioning of immune cells and this contributes to the development of inflammatory and autoimmune diseases [137,138].

Several independent groups have investigated the relationship between mitochondrial mutations and mitophagy. The role of m.12338T>C mutation in decreased mitophagy was demonstrated by reduced levels of autophagy protein light chain 3 and accumulation of autophagic substrate p62 in the in mutant cybrids as compared with control lines. These data demonstrated the direct link between mitochondrial dysfunction caused by complex I mutation and apoptosis or mitophagy [139]. In Leber’s hereditary optic neuropathy (LHON), a classical mitochondrial disease caused by mutations in the mtDNA encoding complex I subunits, autophagy was found to be impaired, resulting in reduced clearance of dysfunctional mitochondria contributing to cell death. Moreover, pharmacological activation of autophagy allowed selective clearance of damaged mitochondria, which improved the overall cell survival in LHON cell models. Therefore, impaired mitophagy appears to be an important pathophysiological mechanism linking oxidative stress with LHON pathogenesis [140].

Another study showed that mtDNA mutations that disrupted mitochondrial function stimulated mitophagy and reduced the regenerative capacities of cardiac progenitor cells [141]. Human fibroblasts carrying the m.13514A> G mutation displayed enhanced mitochondrial quality control through mitophagy. This was associated with a specific suppression of mitochondrial Ca^2+^ uptake, which caused stimulation of the autophagic mechanism through the signal axis of AMP-activated protein kinase [142]. A study conducted on a panel of human cybrid cell lines carrying various pathogenic mtDNA mutations demonstrated the relationship of mtDNA-mediated mitochondrial dysfunction with mitophagy signaling pathways. It was found that loss of mitochondrial transmembrane potential led to the recruitment of prometaphase factors to the mitochondria [143].

The relationship between mitochondrial functions and atherosclerosis-associated mitochondrial mutations was investigated on cybrid cell lines carrying various variants of the mtDNA obtained from atherosclerotic patients. The identified mtDNA variants associated with increased mitophagy (as assessed by LAMP gene expression) were: m.3336T>C, m.3256C>T, m.5178C>A, m.3336T>C, m.3256C>T, and m.5178C>A [110].

## 10. The Role of Mitophagy in the Innate Immunity

The relationship between mitophagy and innate immunity is discussed in recent review articles [84,97,144,145,146,147]. The interaction between mitophagy and innate immunity is crucial for the host’s protective response to infection. An innate immune response to pathogenic microorganisms can affect mitochondrial homeostasis including the dynamics of mitochondria or even disrupt their functions [147]. In case of infection or inflammation, the regulation of mitophagy and innate immunity can be combined to stimulate the host’s antimicrobial response and prevent excessive inflammation. The PINK1 / Parkin pathway plays a key role in the activation of mitophagy and regulates innate immune responses during infection. Dysregulation of mitophagy can cause excessive activation of inflammasomes [97].

**Figure 10:**
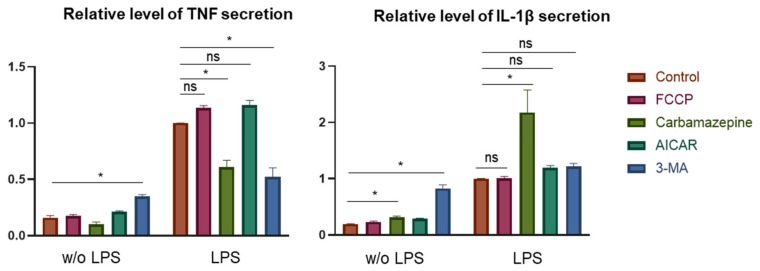
The effect of mitophagy modulators on the ability of human monocytes being activated in response to LPS. Monocytes were isolated from patients using magnetic CD14+ separation. Isolated cells were placed into 24-well plates (106 cells per 1ml of serum free X-VIVO media) with or without mitophagy modulators and had been incubating with or without 1 µg/mL of LPS for 24h. Then secretion level of TNF and IL-1β was measured using ELISA. The values of cytokine secretion by LPS-treated monocytes were taken as 1. Statistical analysis of the results of three independent experiments was carried out using the IBM SPSS Statistics 21 software package. Significance *p* < 0.05 is marked with an asterisk and estimates the difference between Control and added mitophagy modulators according to the results of t-test for paired samples. Ns – no significance, *p* > 0.05. Mitophagy modulators: FCCP, Carbonyl cyanide-4-(trifluoromethoxy) phenylhydrazone, 10-7 M; Carbamazepine, 8 µg/mL; AICAR, 5-aminoimidazole-4-carboxamide 1-β-D-ribofuranoside, 0.3 mM; 3-MA, 3-Methyladenine, 2.5 mM.

The effect of mitophagy modulators on the ability of human monocytes to respond to pro-inflammatory stimulation was studied. Four mitophagy modulators were used (Figure 10). None of the modulators suppressed the secretion of proinflammatory cytokines TNF and IL-1β, but 3-methyladenine and carbamazepine increased it. When stimulated with LPS, these modulators suppressed TNF secretion, but IL-1β secretion was increased by carbamazepine.

Thus, modulation of mitophagy can affect the inflammatory response of innate immunity. Further research should establish a link between defective mitophagy and chronification of inflammation.

## 11. Possible Role of mtDNA Mutations in Chronification of Inflammation

**Figure 11:**
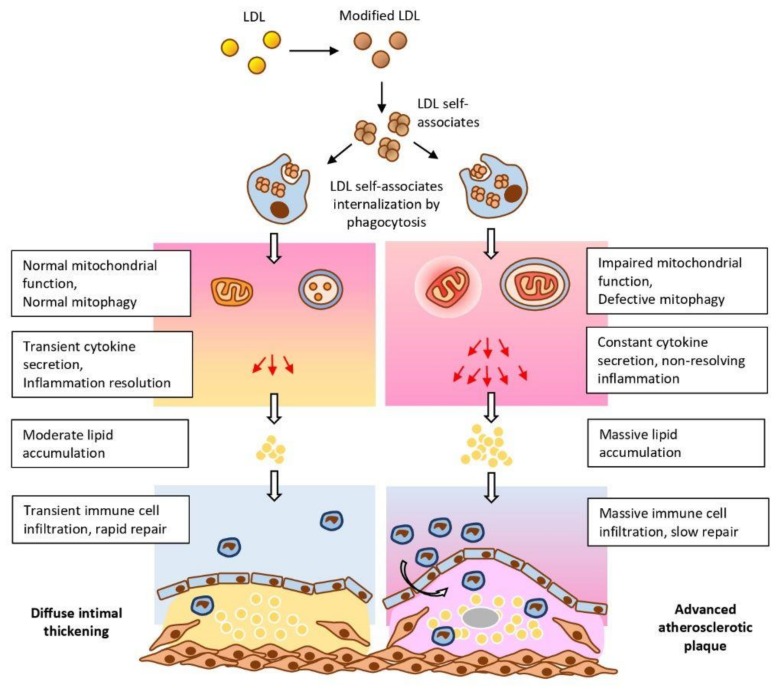
Impaired mitochondrial function and deficient mitophagy promote atherosclerotic lesion formation.

A model that could link the mtDNA mutation-induced mitochondrial dysfunction and deficient autophagy may help understanding of the importance of these events in chronification of inflammation and identifying potential points of therapeutic intervention. Based on the available data discussed above, we propose a plausible mechanism, according to which, phagocytosis stimulation by circulating large associates of modified LDL activate the pro-inflammatory response of the innate immunity system (Figure 11). According to this hypothesis, atherogenic modified LDL circulating in the blood of atherosclerotic patients induces lipid accumulation in the arterial wall cells [148]. Modified LDL particles form self-associates that penetrate the cell by nonspecific phagocytosis, stimulation of which by LDL associates activates the pro-inflammatory response of macrophages in the form of secretion of inflammatory cytokines [149]. Secretion of cytokines leads to increased accumulation of intracellular lipids [31]. If the innate immunity functions normally, the pro-inflammatory reaction resolves rather quickly and further lipid accumulation does not occur. However, when macrophages contain mtDNA mutations, the pro-inflammatory response does not arrest, but rather intensifies with each repeated pro-inflammatory stimulation. The cause of mitochondrial disfunction may be associated with defective mitophagy. Local inflammation in the vascular wall becomes chronic and accompanied by uncontrolled lipid accumulation giving rise to an atherosclerotic lesion. Another intriguing possibility is that cells may recognize the dysfunctional mitochondrion as a pathogen that presents foreign epitopes, therefore triggering the immune response. This may be a consequence of the bacterial origin of mitochondria, due to which defective mitochondria could be recognized by immune cells as pathogens triggering the innate immunity response [150].

The proposed concept allows speculation that atherogenesis is due to two errors made by the cell of the arterial wall. The first one is that the cell perceives the associates of modified LDL as a pathogen that is taken up by phagocytosis, which causes an inflammatory response and the accumulation of intracellular lipids, which in turn is a trigger of atherogenesis at the cellular level. The second is that due to mutations, the mitochondria becomes dysfunctional and due to a defect in mitophagy, the cell cannot free itself from this mitochondrion and perceives it as a bacterium-like pathogen. This triggers an ongoing inflammatory signaling that may lead to inflammasome activation. Mitochondrial dysfunction may be linked to TLR and NLRP3 signaling, with mitochondria therefore playing a key role in the sterile inflammation. Together, these processes result in chronification of inflammation. Such a development may be characteristic not only of atherosclerosis, but of any pathology associated with chronification of inflammation. The specificity of atherosclerosis is that the main triggers of the immune response are not PAMPs or DAMPs but rather, modified LDL. Future studies should investigate the mechanisms of sterile inflammation in atherosclerosis in more detail, paying special attention to mitochondrial dysfunction.

## 12. Future Directions

The important role of mitochondrial mtDNA mutations in the development of various chronic human diseases is currently beyond any doubt. Moreover, mtDNA mutations may serve as important disease-modifying factors. However, outlining the direct links between certain mtDNA variants and pathophysiological features is challenging due to genetic heterogeneity of the mitochondria, the pleiotropic nature of mitochondrial dysfunction input in the overall pathology, and the lack of knowledge about relevant mtDNA variants, although the list of different mtDNA mutations associated with human diseases is constantly growing. Nevertheless, the results available to date position mitochondria as likely therapeutic targets for treatment of such diseases as cancer, chronic inflammatory states, and atherosclerosis. Selective mitochondria-targeting drugs, such as mitochondrial antioxidants, are being tested in preclinical and clinical trials [151,152]. Moreover, certain mtDNA mutations are being currently explored as diagnostic markers [152]. Future studies will improve our understanding of the mechanisms of mitochondrial involvement in the pathological processes. It is likely that studies of deficient mitophagy and the role of certain mtDNA mutations in its development will deliver promising results.

## 13. Conclusions

Clarification of the causes of the transition of acute to chronic inflammation (chronification of inflammation) can reveal the mechanisms of diseases that make up to 80% of morbidity and mortality, as well as explain the causes of aging. Atherosclerosis is one of the most important inflammatory pathologies. The cellular mechanisms of atherogenesis are based on the inflammatory response of innate immunity arising locally or focally. The trigger for this response appears to be modified LDL. With the quick resolution of inflammation, atherosclerotic changes in the arterial wall do not occur. However, a violation of the response of innate immunity can lead to chronification of local inflammation and, as a result, to the formation of an atherosclerotic lesion. Mitochondria dysfunctions are the cause of innate immunity disorders in the case of atherosclerosis and many other pathologies. Some mitochondrial dysfunctions may be due to mutations in mitochondrial DNA. Mitochondrial mutations have been found that can lead to defective mitophagy. The regulatory role of mitophagy in the immune response has been shown in recent studies. We suggest that it is defective mitophagy as a result of mutations in mitochondrial DNA that can cause innate immunity disorders leading to chronification of inflammation.
